# Blood Immunometabolism Correlates with Exercise Intolerance in Older Adults with Heart Failure

**DOI:** 10.1093/geroni/igaf122.784

**Published:** 2025-12-31

**Authors:** Philip Kramer, Zhengrong Gao, Iris Leng, Dalane Kitzman

**Affiliations:** Wake Forest University School of Medicine, Winston-Salem, North Carolina, United States; Wake Forest University School of Medicine, Winston-Salem, North Carolina, United States; Wake Forest University School of Medicine, Winston-Salem, North Carolina, United States; Wake Forest University School of Medicine, Winston-Salem, North Carolina, United States

## Abstract

Heart failure with preserved ejection fraction (HFpEF) is a common and costly geriatric syndrome, causing exercise intolerance in over three million new Americans every year. Blood bioenergetic profiling has been used to assess systemic metabolic health and immunometabolism in older individuals with mobility impairments and other age-related conditions. To test whether the immunometabolism of CD15+ neutrophils and CD14- /CD61- lymphocytes correlate with exercise intolerance, as measured by 6-minute walking distance, we obtained blood from 23 individuals with HFpEF (Avg. 72 YOA, 74% female) and assessed bioenergetics using Seahorse with and without immune activation by phorbol 12-myristate 13-acetate (PMA). PMA-stimulated oxidant production in neutrophils was significantly and positively correlated with walking distance (r = 0.47, p = 0.04). However, the percent of neutrophils in the blood of these subjects tended to be lower in individuals with higher walking speeds (r=-0.33). Lymphocyte ATP-linked respiration tended to be higher in individuals with longer walking distances (r = 0.37), but lymphocyte PMA-stimulated glycolysis, which represents the ability of lymphocytes to switch fuel type upon activation, was not correlated. Older age (60-88) tended to lower walking distance (r=-0.33), neutrophil oxidant production (r=-0.32), lymphocyte ATP-linked respiration (r=-0.21), and significantly lower PMA-stimulated lymphocyte glycolysis (r=-0.45, p = 0.03). These data suggest neutrophil and lymphocyte immunometabolism may serve as live-cell bioenergetic biomarkers for exercise intolerance and aging. Here we report preliminary data on the first subjects in an ongoing study which will help elucidate the role of neutrophil oxidant production and immunometabolism on heart and skeletal muscle function in older patients with HFpEF.

